# Effect of Temperature on the Prevalence of *Saccharomyces* Non *cerevisiae* Species against a *S. cerevisiae* Wine Strain in Wine Fermentation: Competition, Physiological Fitness, and Influence in Final Wine Composition

**DOI:** 10.3389/fmicb.2017.00150

**Published:** 2017-02-07

**Authors:** Javier Alonso-del-Real, María Lairón-Peris, Eladio Barrio, Amparo Querol

**Affiliations:** ^1^Departamento de Biotecnología de los Alimentos, Grupo de Biología de Sistemas en Levaduras de Interés Biotecnológico, Instituto de Agroquímica y Tecnología de los Alimentos (IATA)-CSICValencia, Spain; ^2^Departament de Genètica, Universitat de ValènciaValència, Spain

**Keywords:** *Saccharomyces* species, competition, wine fermentation, temperature, fitness, wine composition

## Abstract

*Saccharomyces cerevisiae* is the main microorganism responsible for the fermentation of wine. Nevertheless, in the last years wineries are facing new challenges due to current market demands and climate change effects on the wine quality. New yeast starters formed by non-conventional *Saccharomyces* species (such as *S. uvarum* or *S. kudriavzevii*) or their hybrids (*S. cerevisiae* x *S. uvarum* and *S. cerevisiae* x *S. kudriavzevii*) can contribute to solve some of these challenges. They exhibit good fermentative capabilities at low temperatures, producing wines with lower alcohol and higher glycerol amounts. However, *S*. *cerevisiae* can competitively displace other yeast species from wine fermentations, therefore the use of these new starters requires an analysis of their behavior during competition with *S. cerevisiae* during wine fermentation. In the present study we analyzed the survival capacity of non-*cerevisiae* strains in competition with *S. cerevisiae* during fermentation of synthetic wine must at different temperatures. First, we developed a new method, based on QPCR, to quantify the proportion of different *Saccharomyces* yeasts in mixed cultures. This method was used to assess the effect of competition on the growth fitness. In addition, fermentation kinetics parameters and final wine compositions were also analyzed. We observed that some cryotolerant *Saccharomyces* yeasts, particularly *S. uvarum*, seriously compromised *S. cerevisiae* fitness during competences at lower temperatures, which explains why *S. uvarum* can replace *S. cerevisiae* during wine fermentations in European regions with oceanic and continental climates. From an enological point of view, mixed co-cultures between *S. cerevisiae* and *S. paradoxus* or *S. eubayanus*, deteriorated fermentation parameters and the final product composition compared to single *S. cerevisiae* inoculation. However, in co-inoculated synthetic must in which *S. kudriavzevii* or *S. uvarum* coexisted with *S. cerevisiae*, there were fermentation performance improvements and the final wines contained less ethanol and higher amounts of glycerol. Finally, it is interesting to note that in co-inoculated fermentations, wine strains of *S. cerevisiae* and *S. uvarum* performed better than non-wine strains of the same species.

## Introduction

Wine is the product of complex interactions among yeast, bacteria and other fungi that begin in vineyards and continue with the fermentation process. Different yeast species are predominant on the surface of grape skins and in the winery environment (Sabate et al., 2002; Albergaria and Arneborg, 2016), and *S. cerevisiae* is recognized as being the main microorganism responsible for this process (Pretorius, 2000). However, other *Saccharomyces* species (*Saccharomyces* non-*cerevisiae* yeasts, SNC) may play an important role in wine fermentation under certain conditions. In this way *S. uvarum* is less frequent than *S. cerevisiae* in wines, but appears to be predominant in European wine regions with an oceanic climate where wine fermentations are performed at lower temperatures; e.g., the Basque Country, Spain (Rementeria, 2003), Alsace, France (Demuyter et al., 2004), Val de Loire, Sauternes, and Jurançon in France (Naumov et al., 2000), Valpolicella, Italy (Torriani et al., 1999), Tokaj in Hungary and Slovakia (Sipiczki et al., 2001; Naumov et al., 2002; Antunovics et al., 2005), and Yalta, the Ukraine (Naumov and Nikonenko, 1987). *S. paradoxus* is a natural species worldwide distributed with a fortuitous presence in vineyards and fermentation processes (Valero et al., 2007). However, some strains of this species have been described as predominant in Croatian vineyards (Redžepović et al., 2002), and exhibit interesting enological properties.

The fermentations conducted by natural interspecific *Saccharomyces* hybrids, such as *S.cerevisiae* × *S.kudriavzevii* and *S.cerevisiae* × *S.uvarum*, have also been described in European wine regions with oceanic and continental climates (northern Spain, Alsace, Germany, Switzerland, Austria, Croatia, Hungary, and Moldavia), close to the northern limit of grapevine distribution (Masneuf et al., 1998; González et al., 2006; Erny et al., 2012; Peris et al., 2012).

Despite these exceptions, presence of SNC in the final stages of the fermentation process is quite rare. This is because *S. cerevisiae* can competitively displace other yeast species from wine fermentations, both SNC (Arroyo-López et al., 2011; Williams et al., 2015) and non *Saccharomyces* yeasts (Holm Hansen et al., 2001; Pérez-Nevado et al., 2006). Different mechanisms have been proposed to explain the higher competing capability of *S. cerevisiae* compared to non *Saccharomyces* yeasts which, in most cases, are not mutually exclusive, but complementary.

The vigorous fermentative capacity of *S. cerevisiae* yeasts in both the presence (Crabtree effect) and absence of oxygen, has been recognized as the main strategy to outcompete other microbial species present in must. *S. cerevisiae* consumes sugar resources faster, and the ethanol and CO_2_ produced during fermentation can be harmful or less tolerated by their competitors. Once competitors are overcome, *S. cerevisiae* can then use accumulated ethanol as a substrate for aerobic respiration. This ecological strategy is called (ethanol) make-accumulate-consume (Thomson et al., 2005; Piškur et al., 2006), and provides a selective advantage to *S. cerevisiae* to outcompete other microorganisms. Different non *Saccharomyces* yeast, as well as bacteria, have also been proven to be very sensitive to the killer peptides or toxic compounds produced by *S. cerevisiae* (Pérez-Nevado et al., 2006; Albergaria et al., 2010; Branco et al., 2014; Wang et al., 2015, 2016), which may play a key role during competition. Finally, the higher *S. cerevisiae* cell density has also been postulated as being an important factor that contribute to the exclusion of non *Saccharomyces* yeasts (Holm Hansen et al., 2001; Nissen et al., 2003, 2004; Nissen and Arneborg, 2003; Arneborg et al., 2005).

Other *Saccharomyces* species share very similar physiological properties with *S. cerevisiae* and, hence, similar ecological strategies. However, wine *S. cerevisiae* yeasts show better adaptation to survive under the stressful environmental conditions occurring during alcohol fermentation; e.g., high concentrations of sugar or ethanol, low pH and nutritional depletion, which provides them with a competitive advantage (Albergaria and Arneborg, 2016).

Another important advantage of *S. cerevisiae* on SNC species is its efficient growth at a wide range of temperatures, especially at higher temperatures (32°C). This has also been considered an important trait that explains its dominance during wine fermentation (Salvadó et al., 2011a). Goddard (2008) also observed that *S. cerevisiae* is even able to significantly increase the environmental temperature during vigorous fermentation. Arroyo-López et al. (2011) also demonstrated that *S. cerevisiae* was able to outcompete *S. kudriavzevii* even at temperatures that are more suitable to the latter (Salvadó et al., 2011b). However, *S. paradoxus* has been shown to be present during grape fermentation processes when competing with *S. cerevisiae* at both 22 and 30°C (Williams et al., 2015). Therefore, very little is known about the behavior of other SNC in competition with *S. cerevisiae* in winemaking environments at low temperatures.

In the Twenty-First century, the wine industry must respond to the challenges posed by both new consumers' demands and changes in grape composition and properties due to climate change. Consumers demand products with lower alcohol content and fruitier aromas, which lead winemakers to lower fermentation temperatures, as far as 10–12°C, to preserve aroma compounds in wines. Climate change influences grape must characteristics (acidity, content in sugars or tannins, etc.), which has an impact on final product quality. Also due to climatic change there is a gap between the maturity according to sugar content and the maturity of the phenolic compounds of the grape. Therefore, sugar concentration in musts reaches higher levels, which leads to wines with higher ethanol content.

These facts strongly challenge the quality and acceptance of the final product which leads to the necessity of improvements in oenological practices, among which the development of new yeast starters adapted to the new imposed conditions are of chief importance. Previous studies have shown that unconventional *SNC* yeast species, such as *S. kudriavzevii* and *S. uvarum*, could be good candidates to achieve those goals. This is because they exhibit good fermentative capabilities at low temperatures (Salvadó et al., 2011b), produce wines with lower alcohol and higher glycerol amounts (Arroyo-López et al., 2010; Oliveira et al., 2014; Pérez-Torrado et al., 2016), and contribute with good aromatic profiles (Gonzalez et al., 2007; Lopandic et al., 2007; Díaz-Montaño et al., 2008; Gamero et al., 2011, 2013, 2014; Stribny et al., 2015). As well as *S. uvarum* and *S. kudriavzevii*, we also included *S. paradoxus*, the closest species to *S. cerevisiae* among those of the *Saccharomyces* genera, which has been already tested for its fermentative capacity as we mentioned above; and *S. eubayanus*, the cryotolerant and recently discovered parental of lager yeast, found in natural fermented beverages from indigenous South American communities (Rodríguez et al., 2014), which makes it a good candidate for screening new properties that might increase the diversity of current commercial wines. Yet despite their potential, these species may have difficulties in competing at the industry level with *S. cerevisiae*, which in most of the cases exhibits better ethanol resistance and the ability to ferment at higher temperatures.

In the present study, we analyzed the survival capacity of SNC in competing with *S. cerevisiae* during fermentation at different temperatures to identify those traits that influence their competitive capabilities and to evaluate their industrial potential. Whereas, genetic markers are the standard to differentiate *Saccharomyces* strains in a complex culture, a quantitative PCR (QPCR)-based approach was designed to avoid them and their possible effect on gene expression or relative fitness. This approach consists on a relative quantification of the proportion of cells based on the QPCR amplification of a gene with species-specific primers using total DNA isolated from a mix of two strains.

## Materials and methods

### Yeast strains

Seven different *Saccharomyces* strains were used in our experiments. We chose a commercial strain, T73 (Lalvin T73 from Lallemand Montreal, Canada), as our wine *S. cerevisiae* representative. We also included YPS128, a *S. cerevisiae* strain isolated from Pennsylvania woodlands; *S. paradoxus* strain 54, isolated from Croatian vineyards; two *S. uvarum* strains, BMV58, selected in our laboratory and commercialized for winemaking (VELLUTOBMV58™ from Lallemand), and CECT12600, isolated from a non-fermented beverage (*mistela*) in Alicante, Spain; *S. eubayanus* strain NPCC1292 is a natural isolate from North Patagonian *Mudai*, traditional fermentation made with *Araucaria araucana* seeds; and *S. kudriavzevii* strain CR85, a natural isolate from oak tree bark in Agudo, Ciudad Real, Spain.

### Synthetic must fermentations

For all our experiments, fermentations were performed in 3x or 6x replicates in 250 mL flasks that contained 200 mL of synthetic must (SM), which is frequently used in microvinification experiments (Rossignol et al., 2003), with 100 g/L of glucose and 100 g/L of fructose.

To assess the relative growth of *S. cerevisiae* and other *Saccharomyces* species under winemaking conditions, we performed competition experiments in which we measured the relative amount of both strains in co-cultures. We included a *S. cerevisiae* strain, either T73 or YPS128, and a non *cerevisiae* one, in all these experiments and measured their relative abundance at different fermentation times. As controls, we monitored the growth of each strain in monocultures under the same conditions as the competitions experiments. Overnight precultures were grown in YPD medium at 25°C. Afterward must was inoculated with the corresponding yeast strain to reach an initial concentration of 10^6^ cells/mL, and was incubated at a fixed temperature (8, 12, 20, or 25°C) with agitation at 100 rpm during fermentation.

Cell samples were collected at several time points during fermentation and kept at −20°C for the subsequent total DNA isolation, used for the QPCR analysis, as described below. Cell counting was carried out in a Neubauer chamber to determine cell density at every sampling point. Growth curves were obtained by considering cell density and the proportion of competing strains given by the QPCR data.

Müller valves were used to monitor fermentation stage through weight loss, until it reached a constant weight, when it was considered to be over. At this point, samples of supernatant were kept at −20°C for further analyses.

### Primer design

**Figure 1:**
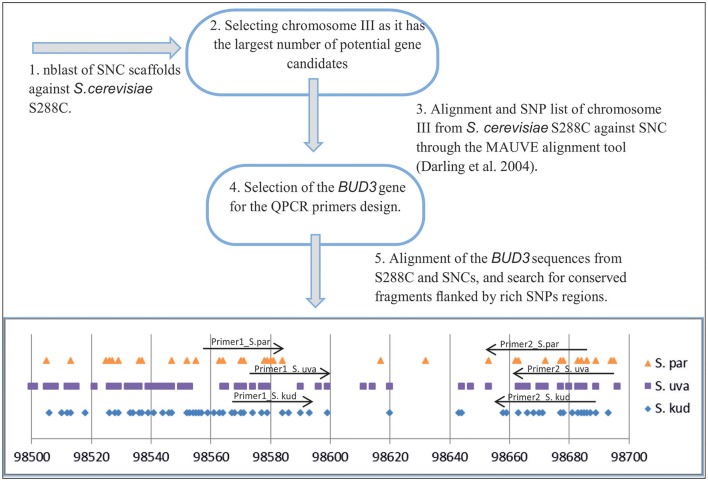
**Scheme used for QPCR primers design**.

Alignments of homologous chromosomes from *S. cerevisiae* S288c, *S. paradoxus, S. kudriavzevii*, and *S. uvarum* were carried out by the Mauve alignment tool (Darling et al., 2004). Genomic sequences were downloaded from the *Saccharomyces Sensu Stricto Resources* website (Scannell et al., 2011) and *Saccharomyces Genome Database* (Engel, 2013). By way of example, a SNPs map of the gene *BUD3* of *S. paradoxus, S. kudriavzevii*, and *S. uvarum* individually aligned against *S. cerevisiae* is shown in Figure 1. This highly conserved single copy gene was selected to look for strain-specific pairs of primers (Supplementary Table 1). All the resulting amplicons were approximately 100 bp in length and had a similar melting temperature when detected with our *LightCycler 480 II* instrument.

### Specificity of the PCR assays

Total DNA samples were extracted from yeasts as described below. PCRs were carried out in a 20 μL final volume, including 1 μL of the DNA template, 0.25 μM of each primer, 200 μM of each dNTP, 2.5 mM of MgCl_2_, 10X buffer, and 0.75 U of *Taq DNA polymerase* (Takara, Bio, Shiga, Japan). For each case, total DNA from the competitor strain was used as a crossed amplification control.

The PCR program consisted of an initial denaturalization step at 94°C for 5 min, followed by 30 cycles of a denaturalization step at 94°C, an annealing step at either 53 or 54°C for 1 min, and an extension step at 72°C for 10 s, and a final extension step at 72°C for 5 min. PCR products were analyzed by electrophoresis on a 1.5% (w/v) agarose gel stained with *RealSafe*™ nucleic acid staining solution (20,000X) (Chembio Diagnosis Systems, Medford, NY, USA) in 1x TAE buffer, and were visualized under UV light. A 100-bp DNA ladder marker (Invitrogen™, Carlsbad, CA, USA) was used as the size standard.

### DNA extraction and sample preparation

Total DNA samples from all the yeasts were extracted as described elsewhere (Querol et al., 1992). The concentration of the DNA samples was measured in a Nanodrop spectrophotometer ND-1000 (Nanodrop Technologies™, Wilmington, DE. USA) and adjusted to 20 ng/μL.

### qPCR analysis

PCR amplification was performed in a 10 μL final volume that contained 2.5 μL of the DNA template, 1.5 μL MilliQ water, 0.2 μM of each primer, and 5 μL of *LightCycler 480 SYBR Green I Master* (Roche). Reactions were performed in 96-well plates in an *LightCycler 480 (II)* PCR amplification and detection instrument with an initial denaturalization step at 95°C for 5 min, followed by 45 cycles of 95°C for 10 s, either 53 or 54°C for 10 s and 72°C for 4 s. The *C*_*T*_ values were calculated automatically by this instrument.

Plates were always divided into two symmetric halves. In each one, a different reaction mix was used where the pair of primers was specific for one of the two strains. For each half, an internal standard curve was included, made of six serial dilutions of the mixed total DNA from both competing strains in 1:1 proportions, the total DNA from the strain amplified in this half as a positive control, the total DNA from the other strain in competition as a control for cross amplification, and a negative control with PCR grade water instead of the template DNA. Three to six biological replicates were used.

The relative concentration of both strains in each biological replicate was given by the ratio of the means of the technical replicates concentrations calculated by the *LightCycler 480 instrument software 1.5* (Roche Diagnosis, Darmstadt, Germany).

### Method sensitivity

For every competition experiment, the following test was performed to assess the reliability of our method. The mix of cells of the corresponding strains was prepared from overnight GPY precultures in known proportions (10:90, 30:70, 50:50, 70:30, and 90:10). The QPCR analysis was carried out using total DNA extraction samples from the mixes of cells. The relative concentration of both strains in each sample was given by the ratio of the means of the concentrations of the replicates given by the *LightCycler 480 instrument software 1.5* (Roche Diagnosis, Darmstadt, Germany). Three biological replicates were included.

Linear model adjustments were made for the cell proportions estimated with each used pair of primers against the theoretical values, and for all the collected data as a whole. The function *lm()* from R was used for this purpose.

### Relative intrinsic growth rate determination

The intrinsic growth rate (*r*) can be calculated as in a previous work of Williams et al. (2015). Here the same method was followed with some modifications:

(1) $$N_{t}=N_{0}e^{rt}$$

where *N*_*t*_ is cell density at a given time point, *N*_0_ corresponds to the initial cell density, and *t* is the time (in hours) when both strains reached their highest cell density in both competition and monoculture.

The effect that competition has on the involved strains can be assessed as the difference in their intrinsic growth rate in single culture and in competition (Δ = *r*_*single*_ − *r*_*competition*_). For the sake of better clarifying the results, the relative intrinsic growth rate (RΔr = Δr/*r*_*single*_) was determined.

### Growth kinetics parameters

On day 1, the precultures of all the used strains were grown o/n at 25°C in GPY medium. On day 2, cells were harvested by centrifugation, washed, suspended in dH_2_O and diluted to an OD_600_ of 2.7. Next 10 μL from each dilution were pipetted into one well of a 96-well plate, previously filled with 260 μL of SM (10 replicates). Four wells were filled with only sterile SM as a blank for the OD_600_ measurements. Four plates were set, one for each assayed temperature: 8, 12, 20, and 25°C.

OD_600_ was monitored in a SPECTROstar Omega instrument (BMG Labtech, Offenburg, Germany). Frequency of measurements varied according to temperature in order to obtain sufficient data points for a statistically significant adjustment to the reparametrized Gompertz equation proposed by Zwietering et al. (1990), which takes this expression:

(2) y=D×exp {−exp[((μmax×e)/D)×(λ−t)+1]}

where *y* = ln (OD_*t*_/OD_0_), OD_0_ is the initial OD and OD_*t*_ is the OD at time *t, D* is the asymptotic maximum, the equivalent to ln (OD_max_/OD_0_), μ_max_ is the maximum specific growth rate (h^−1^) and λ is the lag phase period (h). An adjustment was made using a nonlinear regression procedure of minimizing the sum of the squares of the difference between the experimental data and the fitted model. This was done using version 7.0 of the Statistica software (Stat-Soft, Inc., Tulsa, OK, USA).

Strains were tested for the significant differences among them with an ANOVA using the one-way ANOVA module of the Statistica 7.0 software. Growth parameters μ_max_ and λ were introduced as dependent variables. Means were grouped using the Tukey HSD test (α = 0.05).

### Correlation of relative intrinsic growth rate and growth kinetics parameters

Linear regression models (y = Ax_1_ + B and y = Ax_2_ + B) were constructed, where y = *R*Δ*r* for the non *cerevisiae* strain, x_1_ = (μ_max_competitor__ − μ_max_*S. cerevisiae*__)/μ_max_competitor__ (*R*Δμ) and x_2_ = (λ_*S. cerevisiae*_ − λ_competitor_)/λ_competitor_ (*R*Δλ). This was done using the R function *lm* (R Core Team, 2015).

### HPLC analysis

Residual sugars (glucose and fructose), glycerol, ethanol and acetic acid from the fermentation end point samples were determined by HPLC (Thermo Fisher Scientific, Waltham, MA. USA) using a refraction index detector and a HyperREZTM XP Carbohydrate H+ 8 μm column (Thermo Fisher Scientific) equipped with a HyperREZTM XP Carbohydrate Guard (Thermo Fisher Scientific). Samples were diluted 3-fold, filtered through a 0.22-μm nylon filter (Symta, Madrid, Spain) and injected in duplicate. The analysis conditions were: eluent, 1.5 mM of H_2_SO_4_; 0.6 ml min-1 flux and a 50°C oven temperature.

### Statistical analysis of the fermentation kinetics and HPLC results

The recorded mass loss of the fermentation flasks correlates with sugar consumption, which was taken into consideration to fit our curve to Gompertz equation (Zwietering et al., 1990) and obtain fermentation parameters *m* (maximum sugar consumption rate, g L^−1^ h^−1^), *l* (lag phase period, h) and *t*_90_ (time taken to consume 90% of sugars, h) as in Pérez-Través et al. (2014).

Fermentations were tested for the significant differences among them with an ANOVA using the one-way ANOVA module of the Statistica 7.0 software. The concentrations of glucose, fructose, glycerol, ethanol and acetic acid obtained by HPLC, and the parameters *m, l*, and *t*_90_ were introduced as the dependent variables. Means were grouped using the Tukey HSD test (α = 0.05). The analysis was performed for each temperature condition used.

## Results

### Specificity and sensitivity of the qPCR assay

Six pairs of primers were designed, one for each strain, except for the *S. uvarum* strains, which share primers. To check for specificity, primers were tested by conventional PCR amplification. Bands of the desired size were observed in all cases. Absence of bands from the PCR reactions of the total DNA isolated from the competitor strain confirmed strain specificity.

**Figure 2:**
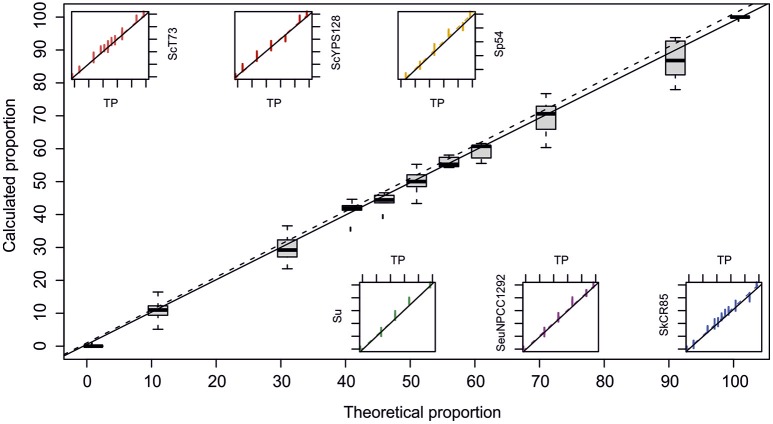
**Calculated relative quantification by QPCR against theoretical values**. Boxplot shows the summary of all the data, while small graphics show the dispersion for each specific pair of primers. Data sets were adjusted to a linear model. Dotted lines represent normal distribution and full lines denote adjustments.

To assess the technique's sensitivity for the relative quantification of different yeast strains in co-culture, mixes of cells in known proportions were prepared for each assayed competition; i.e., our reference *S. cerevisiae* winery strain T73 against *S. kudriavzevii* strain CR85, *S. uvarum* strain BMV58, *S. uvarum* strain CECT12600, *S. paradoxus* strain 54 or *S. eubayanus* strain NPCC1292, and the wild *S. cerevisiae* strain YPS128 against *S. kudriavzevii* strain CR85. The obtained QPCR results about the theoretical proportions can be seen in Figure 2. Data were fitted to linear regression models and coefficients that came very close to the normal for all cases were obtained (Table 1). These results were statistically significant according to the Fisher test (Table 1). Thus, the method is suitable for the quantification of the different *Saccharomyces* strains mixed in a culture.

**Table 1:** **Linear model adjustment results for the calculated relative QPCR quantification (*y*) against the theoretical values (*X*)**.

**Pair of primers**	**A**	**B**	**R2**	***p*-value**
*S. cerevisiae* T73	1.0089	−0.5715	0.9924	<2.2 × 10-16
*S. paradoxus* 54	1.0074	1.2536	0.9829	1.473 × 10-15
*S. eubayanus* NPCC1292	0.9834	1.4564	0.9905	<2.2 × 10-16
*S. uvarum* BMV58/CECT12600	0.9640	1.2897	0.9924	3.655 × 10-15
*S. cerevisiae*YPS128	1.0214	−1.6341	0.9867	<2.2 × 10-16
*S. kudriavzevii* CR85	1.0182	0.5339	0.9905	<2.2 × 10-16
All	10060	0.043	0.9889	<2.2 × 10-16

*A, is the regression coefficient and B, is the error term. p-values are obtained by the Fisher test*.

### Yeast competitions

To assess the effect of competition at low temperature on the intrinsic growth rate (*r*) of the SNC species, we performed a series of fermentations conducted by yeast strains *S. paradoxus* 54, *S. uvarum* BMV58, *S. uvarum* CECT12600, *S. kudriavzevii* CR85 and *S. eubayanus* NPCC1292 in competition with wine *S. cerevisiae* strain T73. We also tested the behavior of wild *S. cerevisiae* strain YPS128 in competition with *S. kudriavzevii* CR85. These competition experiments were performed in batch fermentations of SM at 8, 12, and 20°C. Fermentations at a moderate temperature condition (25°C) were also performed as a control of *S. cerevisiae's* imposition on cryotolerant yeasts. Monoculture fermentations, inoculated with the same strains, were performed as controls under the same conditions.

**Figure 3:**
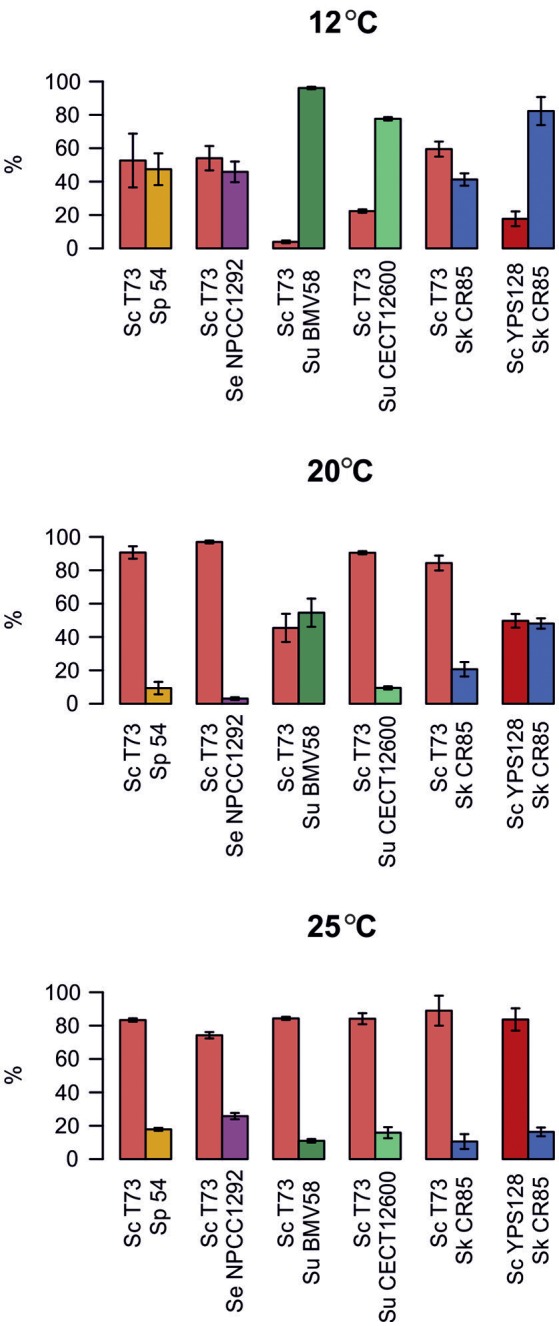
**Presence of both strains from each competition when their highest cell densities were reached**. Values are the mean of three replicates. Error bars represent SD.

Figure 3 shows the percentages of the strains under competition when fermentation reached the stationary growth phase. These results offer an overview of the output of competitions during fermentation at different temperatures. We can see that wine *S. cerevisiae* T73 was able to exclude all the other *Saccharomyces* strains during fermentation at 25°C. At 20°C, T73 also outcompeted all the strains, except for wine *S. uvarum* BMV58, which was present in similar percentages. However, at low temperature, 12°C, T73 co-existed with *S. eubayanus, S. kudriavzevii* and *S. paradoxus*, but was displaced by both the *S. uvarum* strains.

In the competitions between wild strains *S. cerevisiae* YPS128 and *S. kudriavzevii* CR85, YPS128 clearly outcompeted CR85 at 25°C, they co-existed at 20°C, but CR85 certainly dominated at low temperatures.

**Figure 4:**
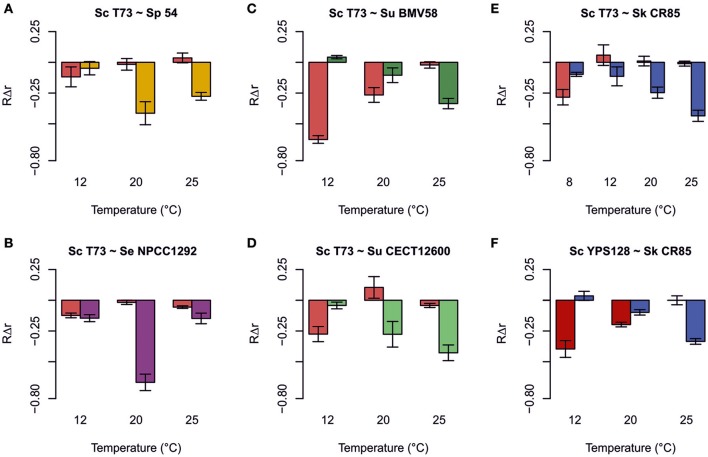
**Relative intrinsic growth rate (RΔr = (*r*_*competition*_ − *r*_*single*_)/*r*_*single*_) caused by the effect of competitions between Saccharomyces cerevisiae T73 and *S*. *paradoxus* 54 (A)**, *S. cerevisiae* T73 and *S. eubayanus* NPCC1292 **(B)**, *S. cerevisiae* T73 and *S. uvarum* BMV58 **(C)**, *S. cerevisiae* T73 and *S. uvarum* CECT12600 **(D)**, *S. cerevisiae* T73 and *S. kudriavzevii* CR85 **(E)**, and *S. cerevisiae* YPS128 and *S. kudriavzevii* CR85 **(F)**. Values are the means of triplicate experiments. Error bars represent SD.

Despite these results being quite explicative about domination during competition, it is interesting to obtain a quantitative measurement of the effect that presence of a particular yeast can have on its competitor's growth. The most suitable indicator of these effects is the relative intrinsic growth rates (*R*Δ*r*) based on the difference of growth rates when the strain is grown in a mixed culture and in a single culture (Figure 4). It is important to note that there was no significant positive effect on the growth of any strain as a result of the presence of a competitor. For *S. cerevisiae*, these effects were negative at low temperatures, but null or insignificant at high temperatures (Figure 4). For *S. uvarum* and *S. kudriavzevii*, a trend in the opposite direction was noted as the effect was less negative (or insignificant) at low temperatures, and the negative effect increased with temperature (Figures 4C–F). Finally for *S. paradoxus and S. eubayanus*, the strongest negative effect occurred at medium temperature (20°C) (Figures 4A,B).

The comparison made between the performances of strains of the same species, but with different origins, showed that *S. cerevisiae* T73 and *S. uvarum* BMV58 wine strains were considerably less affected by their competitors than the strains with other origins, such as *S. cerevisiae* YPS128 and *S. uvarum* CECT12600 (Figures 4C–F).

Prevalence during fermentation seemed to be clearly related to temperature adaptation. The correlations of growth parameters maximum growth rate and lag phase duration (Table 2) with the relative increment in the intrinsic growth rate were calculated. Positive correlations with R^2^ ~ 0.4 were obtained for both parameters.

**Table 2:** **Growth parameters obtained by the Gompertz equation proposed by Zwietering et al. (1990) for the different strains in SM at different temperatures**.

	**8°C**		**12°C**		**20°C**		**25°C**	
**Strain**	**μ_max_ (h^−1^)**	**λ (h)**	**μ_max_ (h^−1^)**	**λ (h)**	**μ_max_ (h^−1^)**	**λ (h)**	**μ_max_ (h^−1^)**	**λ (h)**
*S. cerevisiae* T73	0.0083±0.0002^c^	247.77±9.15^e^	0.0217±0.0005^b^	82.5652±0.87^c^	0.0602±0.0020^a^	18.6771±0.43^a^	0.1740±0.0060^a^	9.3204±0.62^a^
*S. paradoxus* 54	0.0080±0.0003^b, c^	260.69±9.10^f^	0.0231±0.0011^c^	104.5469±2.38^a^	0.0504±0.0034^c^	25.2771±1.01^c^	0.1387±0.0021^c^	12.7527±1.47^d^
*S. eubayanus* NPCC1292	0.0098±0.0012^d^	143.58±2.47^a^	0.0216±0.0006^b^	63.4613±2.10^e^	0.0471±0.0016^d^	26.5521±0.44^d^	0.1184±0.0022^d^	10.3316±0.18^a, b^
*S. uvarum* BMV58	0.0181±0.0009^f^	147.09±4.09^a^	0.0320±0.0008^e^	66.1784±1.07^d^	0.0609±0.0024^a^	27.7608±0.39^e^	0.1668±0.0214^a, b^	12.1358±1.04^c, d^
*S. uvarum* CECT12600	0.0153±0.0005^e^	160.02±1.81^b^	0.0301±0.0006^d^	71.2131±1.95^b^	0.0516±0.0009^c^	26.2352±0.52^d^	0.1539±0.0049^b^	11.4711±0.30^b, c^
*S. cerevisiae* YPS128	0.0067±0.0003^a^	220.15±7.35^d^	0.0194±0.0003^a^	83.1130±2.23^c^	0.0554±0.0013^b^	23.3442±0.49^b^	0.1560±0.0040^b^	9.5580±0.69^a^
*S. kudriavzevii* CR85	0.0074±0.0002^a, b^	178.10±4.65^c^	0.0199±0.0005^a^	65.0655±2.06^d, e^	0.0437±0.0020^e^	25.8793±0.95^c, d^	0.1023±0.0071^e^	14.5462±0.55^e^

*μ_max_, is maximum growth rate and λ, is lag phase duration. Values are given as mean ± standard deviation. The values followed by different superindexes in the same column are significantly different according to the Tukey HSD test (α = 0.05, n = 10)*.

#### Competitions between *S. cerevisiae* T73 and *S. paradoxus* 54

When competing with *S. paradoxus* strain 54, T73 achieved slightly lower intrinsic growth rate at 12°C compared to a single fermentation. However, at 20 and 25°C, its growth fitness is maintained (Figure 4A). Strain 54 performed normally at low temperature, but was clearly affected at 20 and 25°C (Figure 4A), and was almost totally excluded from fermentation (Figure 3). Although both species were phylogenetically closely related, the wine *S. cerevisiae* strain seemed superior in this competition. Furthermore, it is interesting to note that at all tested temperatures, the dominant strain T73 had a shorter lag phase (λ, Table 2).

#### Competitions between *S. cerevisiae* T73 and *S. eubayanus* NPCC1292

In competition both strains maintained their capability to grow in co-cultures at 12 and 25°C, according to the slight drop in their intrinsic growth rate parameter compared to single cultures. Strikingly at intermediate temperatures, NPCC1292 was clearly outcompeted by T73 (Figure 4B), when its lag phase became noticeably longer (Table 2). Although the intrinsic growth rate of NPCC1292 was only slightly affected at 25°C, this strain was present at a low percentage during fermentation (Figure 3). This can be explained by a low cell density during not only competition, but also during single culture fermentation (data not shown).

#### Competitions between *S. cerevisiae* T73 and *S. uvarum* strains

Here we assessed the competitive adaptation capacity of a wine and a non fermentative *S. uvarum*. Wine *S. uvarum* strain BMV58 competed better at low temperatures (12 and 20°C), and severely affected T73 growth. This effect reverted as temperature rose. We can see that T73 shows a clear advantage at 25°C (Figure 4C).

To test whether the same trend could be observed with a non wine strain, we performed the same experiment using strain *S. uvarum* CECT12600. The behavior of the differential intrinsic growth rates was similar, but in this case *S. uvarum* CECT12600 obtained lower values and had a less intense effect on T73 (Figure 4D) than BMV58, which showed better competitive fitness in fermentative environments.

Finally, it is important to remark that *S. cerevisiae* T73 had a shorter lag phase (λ) than *S. uvarum* BMV58 during the competitions at 20 and 25°C (Table 2), but at similar maximum growth rates (μ_max_), and BMV58 was able to co-exist with T73 during the competition at 20°C, but not at 25°C (Figure 4). At 12°C, both *S. uvarum* strains had a shorter lag phase and higher maximum growth rates than *S. cerevisiae* (Table 2), and were dominant during fermentation (Figure 3).

#### Competitions between *S. kudriavzevii* CR85 and *S. cerevisiae* strains

Wine strain *S. cerevisiae* T73 is not affected by most temperature conditions when competing with *S. kudriavzevii*. However, at 8°C, a clear negative effect on the relative intrinsic growth rate (*r*) on T73 can be observed. *S. kudriavzevii* CR85 was always affected by presence of T73, although its impact was softer at 8°C and *S. kudriavzevii* became more competitive (Figure 4E).

To test if T73 resistance during competition, even at a very low temperature, was to some extent dependent on its better adaptation to fermentation environments, the wild *S. cerevisiae* strain YPS128, isolated from an oak bark, was used in the competitions assays with *S. kudriavzevii*. Figure 4F shows noticeable differences in the competition at 12°C, where CR85 clearly outcompetes YPS128, and it exhibited an intrinsic growth rate that was markedly affected. In the competitions at 20°C, in which *S. kudriavzevii* predominated (Figure 3), YPS128 underwent a greater negative effect (Figure 4). Contrarily at 25°C, CR85 was clear at a disadvantage (Figure 4F). Therefore, the fermentative origin of the *S. cerevisiae* yeasts seems to correlate with better performance in fermentation.

The growth parameters from Table 2 could explain most of these results. At 8°C, when *S. Kudriavzevii* outcompeted *S. cerevisiae* T73, the winner (Figure 3) had a higher maximum growth rate and a shorter lag phase. At 12°C, *S. kudriavzevii* presented a shorter lag phase than both the *S. cerevisiae* strains, and a higher maximum growth rate than the wild *S. cerevisiae* strain, which was clearly affected under these conditions (Figure 4F). *S. cerevisiae* wine strain T73 had a clearly higher μ at 12°C, which could be the reason why T73 became dominant as fermentation continued (Figure 3). At 20°C, both the *S. cerevisiae* strains already exhibited better growth capabilities in synthetic must (Table 2), but there were clear differences in their performance during the competition against *S. kudriavzevii* as the wine strain was a much better competitor than the wild strain (Figures 3, 4E,F).

#### Correlation between growth parameters and competitive advantage

We assessed whether there was any correlation between the fact of having better growth parameters in single culture and the imposition during our competition experiments (Table 2). Linear correlations between RΔr and RΔμ or RΔλ were obtained, with *R*^2^-values of 0.40 and 0.42, respectively. Significance was tested by the Fisher's test, and the resulting *p*-values were 0.005158 and 0.003681, respectively.

**Figure 5:**
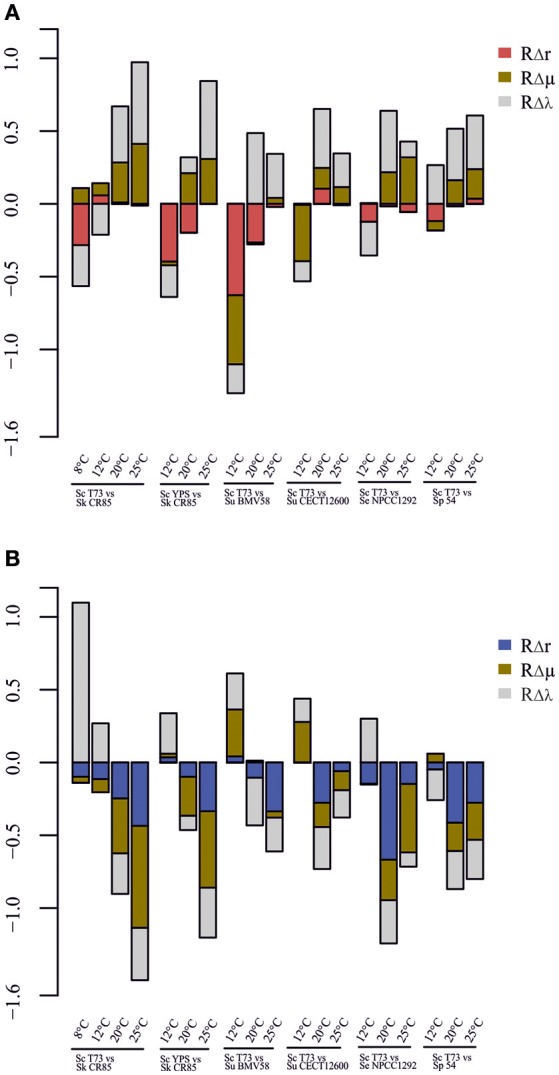
**Comparative of performance in competition and growth kinetics parameters in single culture for *S. cerevisiae* (A)** and competitor strains **(B)**.

A graphical summary of the three parameters used in the analysis for each competition is depicted in Figure 5. In most cases, low RΔr values corresponded to low RΔμ and RΔλ. This indicates that a more affected strain during co-fermentations exhibits worse growth parameters in single culture than its competitor (Figure 4, Table 2).

There are some exceptions however, as already mentioned above, such as the competition between T73 and BMV58 at 20°C, at which *S. uvarum* had a similar RΔμ and a notably worse RΔλ value, but competition had a remarkably negative effect on *S. cerevisiae*. Interestingly, Figure 5 shows comparatively slight RΔμ or RΔλ differences together with extreme RΔr values (T73-NPCC1292, 20°C), and vice versa (T73-NPCC1292, 25°C). This indicates that co-culture fermentations may be influenced by other competitive growth strategies.

### Influence of competition on fermentation parameters

Yeast characterization as wine fermenters must include aspects like the ability to consume all the sugars present in must at a suitable pace, or the capability to produce a wine with high quality standards according to consumer demands. Table 3 includes different fermentation kinetic parameters: maximum sugar consumption rate (*m*) and fermentation lag phase (*l*), inferred from mass loss during fermentation, as well as the time taken to consume 90% of the initial sugar content (*t*_90_). Final product composition is also a key factor. Thus, we measured glucose, fructose, glycerol and ethanol concentrations at the end of fermentation, that is, when no mass loss was observed (Table 4). This data set is useful to determine the mixed yeast cultures that could potentially improve some wine characteristics.

**Table 3:** **Kinetics parameters of fermentations m is the maximum sugar consumption rate, l is the fermentation lag phase duration, and t90 is the time employed to consume 90% of the sugars present in the initial must**.

	**12°C**			**20°C**			**25°C**		
**Fermentation**	**m (g L^−1^ h^−1^)**	**l (h)**	**t_90_ (h)**	**m (g L^−1^ h^−1^)**	**l (h)**	**t_90_ (h)**	**m (g L^−1^ h^−1^)**	**l (h)**	**t_90_ (h)**
**T73**	**0.31 ± 0.01^a, b^**	**91.71 ± 1.10^a, b, c^**	**472.92 ± 5.36^a, b, c^**	**0.90 ± 0.06^d, e^**	**26.70 ± 1.24^a, b^**	**171.24 ± 10.73^a, b^**	**1.14 ± 0.17^b, c, d^**	**6.42 ± 1.69^b, c^**	**149.22 ± 17.43^b, c^**
54	0.28±0.01^a, b^	131.64±2.41^e^	589.40±7.35^c, d^	0.82±0.05^b, c, d, e^	33.96±3.05^c, d^	181.52±8.54^a, b^	1.51±0.33^d, e^	9.37±2.47^b, c, d^	122.13±12.43^a, b^
T73-54	0.31±0.00^a^	97.06±3.10^b, c, d^	583.58±8.43^c, d^	0.86±0.16^b, c, d, e^	31.38±4.25^b, c^	247.05±28.78^c^	0.87±0.01^a, b^	12.71±0.51^d, e, f^	171.69±10.93^c^
NPCC1292	0.21±0.00^a^	76.27±6.64^a, b^	Na	0.59±0.01^a, b^	30.14±0.89^a, b, c^	Na	0.65±0.08^a^	23.46±1.59^h^	Na
T73-NPCC1292	0.27±0.01^a^	79.47±5.87^a, b^	656.23±103.49^d^	1.01±0.08^e^	27.87±0.99^a, b, c^	198.77±6.62^b^	0.91±0.10^a, b^	17.23±1.67^g^	162.83±14.64^c^
BMV58	0.30±0.01^a, b^	85.12±1.58^a, b, c^	505.21±8.14^b, c^	0.71±0.04^a, b, c, d^	85.72±1.50^e^	260.97±9.17^c^	0.89±0.02^a, b, c^	2.09±0.72^a^	179.49±4.74^c^
T73-BMV58	0.49±0.01^c, d^	71.20±2.32^a^	320.76±6.27^a^	0.63±0.03^a, b, c^	84.49±1.58^e^	283.37±17.12^c, d^	1.47±0.14^d, e^	15.86±0.46^f, g^	104.81±7.54^a^
CECT12600	0.50±0.01^d^	92.59±3.77^a, b, c, d^	383.64±26.29^a, b^	1.00±0.15^e^	31.43±2.40^b, c^	172.47±23.94^a, b^	1.68±0.03^e^	21.79±0.16^h^	100.45±2.40^a^
T73-CECT12600	0.38±0.01^b, c^	80.79±6.18^a, b^	421.02±18.23^a, b^	1.00±0.06^e^	23.18±2.15^a^	146.04±7.17^a^	1.72±0.23^e^	15.71±0.63^e, f, g^	99.89±17.82^a^
YPS128	0.27±0.01^a, b^	97.84±9.08^b, c, d^	727.23±5.36^d^	0.88±0.03^c, d, e^	31.39±0.62^b, c^	172.66±4.49^a, b^	1.37±0.03^c, d, e^	11.98±0.40^d, e^	116.24±2.03^a, b^
YPS128-CR85	0.31±0.01^a, b^	96.23±4.05^b, c, d^	489.54±6.61^b, c^	0.88±0.04^c, d, e^	27.71±1.88^a, b, c^	321.89±0.50^d^	1.43±0.07^d, e^	10.37±0.77^c, d^	111.70±8.21^a, b^
CR85	0.32±0.01^a, b^	116.35±2.49^d, e^	502.88±7.59^b, c^	1.02±0.00^e^	38.50±1.16^d^	156.49±0.87^a, b^	0.87±0.07^a, b^	15.68±0.62^e, f, g^	184.99±13.71^b^
T73-CR85	0.52±0.14^d^	103.32±19.91^c, d^	382.17±26.05^a, b^	0.54±0.07^a^	24.15±1.86^a^	259.85±19.33^c^	1.65±0.11^e^	6.17±0.78^b^	98.44±9.57^a^

*Parameters are obtained through an adjustment to Gompertz (Zwietering et al., 1990). Values given as mean±standard deviation of three biological replicates. An ANOVA analysis was carried out. The values followed by different superindexes in the same column are significantly different according to the Tukey HSD test (α = 0.05)*.

**Table 4:** **Chemical composition of the fermented SM obtained through HPLC**.

	**12°C**				**20°C**				**25°C**			
**Fermentation**	**glucose**	**fructose**	**glycerol**	**ethanol**	**glucose**	**fructose**	**glycerol**	**ethanol**	**glucose**	**fructose**	**glycerol**	**ethanol**
**T73**	**0.10 ± 0.04^a, b^**	**6.64 ± 1.04^a, b, c, d^**	**5.09 ± 0.03^a^**	**12.49 ± 0.28^c, d^**	**0.02 ± 0.01^a^**	**4.16 ± 0.60^a, b, c^**	**5.53 ± 0.24^a, b, c^**	**12.47 ± 0.48^d, e^**	**0 ± 0^a, b^**	**0 ± 0^a^**	**5.78 ± 0.08^a, b^**	**12.67 ± 0.10^c, d, e^**
54	0 ± 0^a, b^	2.90 ± 0.33^a, b^	5.03 ± 0.04^a^	11.83 ± 0.10^b, c, d^	0.02 ± 0.02^a^	4.06 ± 2.11^a, b, c^	6.08 ± 0.29^b, c, d^	11.21 ± 0.34^b, c, d^	0.21 ± 0.02^a, b^	8.91 ± 0.31^c^	7.57 ± 0.25^e^	10.76 ± 0.04^a, b^
T73-54	0.51 ± 0.10^a, b^	14.88 ± 0.70^e^	5.06 ± 0.12^a^	12.09 ± 0.28^b, c, d^	0 ± 0^a^	4.30 ± 0.41^a, b, c^	5.95 ± 0.37^b, c, d^	13.19 ± 0.44^e, f^	0.28 ± 0.06^a, b^	8.5 ± 1.94^b, c^	5.92 ± 0.12^a, b, c^	12.25 ± 0.40^b, c, d, e^
NPCC1292	6.66 ± 0.80^c^	41.05 ± 0.50^f^	7.68 ± 0.40^d^	9.69 ± 0.54^a^	2.25 ± 0.24^b^	24.54 ± 2.45^e^	6.66 ± 0.08^d, e^	9.89 ± 0.01^a, b^	2.73 ± 1.20^c^	31.47 ± 5.03^e^	7.46 ± 0.16^e^	10.14 ± 0.23^a^
T73-NPCC1292	0.69 ± 0.58^b^	13.45 ± 5.03^d, e^	5.95 ± 0.64^a, b^	11.40 ± 0.70^b, c, d^	0.35 ± 0.34^a^	9.31 ± 3.09^c, d^	6.76 ± 0.27^d, e^	13.97 ± 0.58^f^	0 ± 0^a^	0.56 ± 0.80^a^	6.14 ± 0.09^a, b, c^	12.70 ± 0.18^e^
BMV58	0 ± 0^a, b^	0.70 ± 0.62^a^	5.15 ± 0.17^a^	11.71 ± 0.46^b, c, d^	0 ± 0^a^	2.93 ± 2.55^a, b^	4.72 ± 0.14^a^	9.51 ± 0.16^a^	0 ± 0^a, b^	4.15 ± 1.36^a, b, c^	7.13 ± 0.20^d, e^	12.27 ± 0.08^b, c, d, e^
T73-BMV58	0 ± 0^a, b^	4.91 ± 1.34^a, b^	5.85 ± 0.05^a, b^	11.83 ± 0.16^b, c, d^	0 ± 0^a^	0 ± 0^a^	5.50 ± 0.01^a, b^	10.97 ± 0.74^a, b, c, d^	0 ± 0^a^	3.59 ± 1.45^a^	5.95 ± 0.14^a, b^	12.64 ± 0.09^c, e^
CECT12600	0.12 ± 0.12^a, b^	9.80 ± 2.94^b, c, d, e^	6.05 ± 0.08^a, b, c^	11.26 ± 0.38^a, b, c, d^	0 ± 0^a^	0 ± 0^a^	5.85 ± 0.08^b, c, d^	12.27 ± 0.07^d, e^	0 ± 0^a, b^	3.11 ± 0.53^a^	6.31 ± 0.31^b, c, d^	11.49 ± 0.45^a, b, c, d, e^
T73-CECT12600	0 ± 0^a^	3.92 ± 1.46^a, b^	6.00 ± 0.42^a, b^	12.44 ± 0.70^c^	0.59 ± 0.23^a^	12.76 ± 2.45^d^	5.31 ± 0.01^a, b^	11.51 ± 0.57^c, d^	0 ± 0^a, b^	1.13 ± 1.13^a^	5.46 ± 0.36^a, b^	11.11 ± 0.24^a, b, d^
YPS128	0.26 ± 0.09^a, b^	8.35 ± 1.08^a, b, c, d, e^	6.88 ± 0.57^b, c, d^	10.87 ± 0.31^b, c, d^	0.26 ± 0.07^a^	8.35 ± 1.08^b, c, d^	6.88 ± 0.57^d, e^	12.03 ± 0.10^c, d, e^	0.01 ± 0.01^a, b^	3.52 ± 0.36^a^	6.06 ± 0.15^a, b, c^	10.78 ± 0.94^a, b^
YPS128-CR85	0 ± 0^a, b^	3.13 ± 0.28^a, b^	7.07 ± 0.07^b, c, d^	11.76 ± 0.74^b, c, d^	0.56 ± 0.36^a^	11.31 ± 4.53^d^	6.63 ± 0.29^c, d, e^	11.78 ± 0.24^c, d, e^	0.14 ± 0.11^ab^	4.31 ± 1.87^a, b, c^	6.17 ± 0.90^a, b, c^	10.35 ± 0.77^a^
CR85	0.0 ± 0^a, b^	4.89 ± 2.81^c, d, e^	7.34 ± 0.23^c, d^	10.87 ± 0.46^a, b, d^	0.01 ± 0.01^a^	4.13 ± 0.31^a, b, c^	7.61 ± 0.94^e^	10.54 ± 1.25^a, b, c^	0.85 ± 0.14^b^	16.35 ± 0.90^d^	6.93 ± 0.51^c, d, e^	10.46 ± 0.46^a^
T73-CR85	0.12 ± 0.17^a, b^	6.42 ± 5.37^a, b, c^	7.24 ± 1.32^c, d^	10.36 ± 1.41^a, b^	0.05 ± 0.75^a^	2.73 ± 3.11^a, b^	5.22 ± 0.51^a, b^	11.44 ± 0.26^c, d^	0.03 ± 0.04^a, b^	3.95 ± 1.28^a, b^	5.35 ± 0.24^a^	11.17 ± 0.72^a, b, c, d^

*Glucose, fructose and glycerol are given in g L^−1^, and ethanol in %. Values are given as mean ± standard deviation of three biological replicates and two HPLC detection runs. An ANOVA analysis was carried out. The values followed by different superindexes in the same column are significantly different according to the Tukey HSD test (α = 0.05)*.

Reference wine strain *S. cerevisiae* T73 is characterized by the production of relatively low glycerol values (5–6 g L^−1^) and high ethanol content (>12 %), as observed in Table 4. It also accomplishes quite a high sugar consumption rate at 20°C (Table 3) and 25°C (Table 3), but a low one at 12°C (Table 3), which is consistent with the temperature adaptation of *S. cerevisiae* to grow at higher temperatures than cryotolerant species *S. uvarum* and *S. kudriavzevii*. In most cases, and according to the ANOVA analysis, its *l* and *t*_90_ belong to the group of the shortest times (Table 3).

Interestingly, some co-cultures improved these fermentation parameters; e.g., T73 with either *S. kudriavzevii* CR85 or *S. uvarum* BMV58 at 12°C increased the *m*, and reduced *t*_90_ (Table 3). At 25°C, the combinations of T73 with *S. kudriavzevii* and *S. uvarum* once again seemed to improve the fermentation kinetics. A reduction of *t*_90_ for the three co-cultures (T73-CR85, T73-BMV58 and T73-CECT12600) was also observed at 25°C (Table 3). These fermentation parameters improved compared to their respective single culture fermentations (Table 3), which is indicative of synergic interactions.

Unlike the 12and 25°C conditions, practically no fermentation parameters or compounds improved at 20°C (Tables 3, 4). The competitions against *S. paradoxus* seemed disadvantageous at 20°C and 25°C, which also occurred when competing with CR85 at 20°C and with NPCC1292 at 25°C (Table 3).

Despite their diverse origins, all the strains were able to complete their fermentations at 25°C except *S. eubayanus* NPCC1292 (Table 4). By the end of the fermentations conducted by this strain, the final product contained large amounts of glucose, and especially fructose. At low and medium temperatures (12 and 20°C) some strains also left a considerable amount of sugars, such as *S. cerevisiae* YPS128 or competences NPCC1292-T73, CECT12600-T73, 54-T73 and CR85-YPS128 (Table 4). Interestingly, most of them were able to ferment all the sugars when cultured alone (Table 4), so this could result in an antagonist effect for these pairs of strains. Moreover, some other parameters also reflected worse performances during co-fermentations, specifically *t90* of T73-NPCC1292 at 12°C, or T73-54 and T73-CECT12600 at 20°C (Table 3).

As previously mentioned, a more profitable interaction is observed for CR85-T73 at low temperatures. At 12°C both strains co-existed during fermentation in similar proportions (Figure 3), which led to a final product with a lower ethanol concentration and a higher glycerol content than those obtained for the fermentations conducted by T73 alone (Table 4). Ethanol concentrations also lowered during co-inoculated fermentations at 20and at 25°C, but the conservative ANOVA test did not support the significance of these differences (Table 4). With the co-cultures of T73 with *S. uvarum*, no significant improvements in the final product composition were observed, although mean glycerol values and ethanol concentrations showed a positive tendency compared to the single *S. cerevisiae* fermentations at 12 and 20°C (Table 4).

## Discussion

### Accurate quantification of different *Saccharomyces* yeasts in co-cultures

Natural auxotrophic or drug-resistant mutants and strains genetically modified with reporter genes have been used to monitor yeast competences in co-cultures or during fermentation, which involves demanding tasks, such as mutant selection or construction, CFU enumeration in selective media, or flow cytometry (Arroyo-López et al., 2011; García-Ríos et al., 2014). Our results indicate that a more straightforward QPCR-based method, which does not require previous cell type separation, is suitable for the relative quantification of yeasts (Figure 2). In fact quantification by QPCR of different organisms in wine, including *Saccharomyces* yeasts, has already been applied (Neeley et al., 2005; Andorrà et al., 2010; Vendrame et al., 2014). However, to our knowledge, this technique has never been used to date to differentiate *Saccharomyces* yeasts during competition in the same environment. This novel approach can be applicable to broaden our knowledge about the ecology of the *Saccharomyces* yeast when competing for the same niche.

### Temperature adaptation affects domination in a fermentative environment

Cryotolerant species *S. kudriavzevii* and *S. uvarum* have been used in this work given the trend to perform fermentation at lower temperatures in the wine industry to preserve the aroma fraction (Torija et al., 2001; Beltran et al., 2002; Gamero et al., 2013; Şener and Yildirim, 2013). Our results clearly show a longer prevalence of these species in fermentations at low temperatures. However, at 25°C, *S. cerevisiae* outgrow them. It can also be drawn from our data that this effect is modulated by the adaptation of strains to different habitats, where wine strains are always more competitive no matter what the temperature. This was observed not only for *S. cerevisiae*, but also for *S. uvarum*. Therefore, adaptation to high sugar environments could be another trait that influences fermentation domination as indicated by Barrajón et al. (2011).

Salvadó et al. (2011b) analyzed the thermotolerance of different *Saccharomyces* species using their growth kinetics parameters as measurable indicators. Growth ability under settled conditions could be considered as a suitable predictor for the imposition of one strain on another in competition. However, previous works (García-Ríos et al., 2014), as well as ours, have revealed that domination of environments is a more complex trait on *Saccharomyces* yeasts. Figure 5 shows that in most cases a higher μ_max_ and a lower λ correlate to greater invulnerability in co-fermentation than the competitor strain. Nevertheless, we observed that the intensity of the effect is widely variable, and in some cases we found that the contrary happens; i.e., *S. eubayanus* NPCC1292 in the competition against *S. cerevisiae* T73 at 12°C, whose growth was affected despite having a shorter lag phase. *S. cerevisiae* YPS128, a strain isolated from oak trees, performed worse than expected against *S. kudriavzevii* CR85 at 20°C. Something similar occurred with the competition between *S. cerevisiae* T73 and *S. uvarum* BMV58 for both wine strains at 20°C: T73 had a noticeably shorter λ and a similar μ_max_, but was clearly wakened by BMV58. Thus, it is conceivable that an interaction among yeasts or their side products takes place as part of the competition mechanism. Whether this means that the presence of toxic compounds targets some specific *Saccharomyces* yeasts, the inhibitory physical contact among them, or some other strategy, is something that needs to be looked at in the future.

### Coinoculation of *Saccharomyces* yeasts can be potentially beneficial for fermentations and final product composition

One of the main goals when studying alternative organisms for their use in food fermentation is achieving new characteristics of interest, that these organisms give rise to. With wine, numerous studies have been carried out with non *Saccharomyces* yeasts, and most have focuses on improving or enriching of aroma profiles, whereas others have focused more on controlling the final product concentration of specific compounds, such as ethanol or acetic acid (Andorrà et al., 2012; Rantsiou et al., 2012; Medina et al., 2013; Contreras et al., 2014; Izquierdo Cañas et al., 2014; Zara et al., 2014; Canonico et al., 2015, 2016; Rodrigues et al., 2016). However, fewer studies have been published about fermentation characterization by combining *Saccharomyces* strains or using uncommon *Saccharomyces* species (Cheraiti et al., 2005; Howell et al., 2006; King et al., 2010; Arroyo-López et al., 2011; Barrajón et al., 2011; Saberi et al., 2012; Williams et al., 2015; Gustafsson et al., 2016). Just as some of these investigations have suggested, our results showed that the final product composition of co-fermented musts cannot always be predicted from those of mono-fermentations. We observed a range of different scenarios: synergic or antagonist effects, as well as simply additive, depending on the strains and the assayed conditions. Nevertheless, we found some promising combinations of a wine *S. cerevisiae* strain with a SNC one; e.g., the remarkable case of the co-inoculation of *S. cerevisiae* T73 and *S. kudriavzevii* CR85 at low temperatures, which improved the efficiency of the process as regards single inoculations by increasing the maximum sugar consumption rate, and which also yielded a final product that contained less ethanol and more glycerol.

From the kinetics point of view, the co-fermentations of our wine *S. cerevisiae* strain with *S. uvarum* also revealed a positive effect, which was more visible at 12 and 25°C. This kind of synergic effect has been observed in previous works, where the addition of fructophilic yeast *S. bombicola* (similarly to *S. uvarum*) led to faster fructose and glucose consumption (Milanovic et al., 2012). In our case it was also noteworthy that the viability of T73 during competition against wine strain BMV58 was negatively influenced. However, against non winery strain CECT12600, it did not diminish, which means that winery strains could be more capable of sensing other yeasts in fermentative media and over-activate sugar consumption to take advantage of them. This hypothesis would also be supported by our results about the maximum sugar consumption rate (*m*) of the co-fermentations carried out by our reference wine strains *S. cerevisiae* T73 with *S. uvarum* or *S. kudriavzevii*. At any rate it is remarkable that reduced *t*_90_ values occurred during the fermentations run at 25°C for the cases of T73-CR85, T73-BMV58 and T73-CECT12600, when SNC strains were present in small proportions. This could be indicating that *S. cerevisiae* T73 responded to interactions during competences by increasing its metabolism.

To summarize, we have confirmed the great capacity of *S. cerevisiae* to dominate fermentative environments at traditional process temperatures (Holm Hansen et al., 2001; Pérez-Nevado et al., 2006; Arroyo-López et al., 2011; Williams et al., 2015). However, some cryotolerant *Saccharomyces* yeasts, particularly *S. uvarum*, can seriously compromise *S. cerevisiae* fitness during competences at lower temperatures, which explains why *S. uvarum* can replace *S. cerevisiae* during wine fermentations in European regions with oceanic and continental climates (Naumov et al., 2000; Sipiczki et al., 2001; Naumov et al., 2002; Redžepović et al., 2002; Rementeria, 2003; Demuyter et al., 2004). From a biotechnological point of view, the application of cryotolerant *Saccharomyces* species as starters for wine fermentation at low temperature could avoid its colonization by undesirable microorganisms that has been reported by other authors (Ciani and Comitini, 2006).

Our results also suggest that adaptation to winemaking establishes noticeable differences in the performance of *Saccharomyces* yeasts when competing during wine fermentation. Thus, profounder research on *Saccharomyces* yeasts' physical and biochemical interactions is necessary to optimize the composition of such starter cultures, which would make them even more interesting for industrial purposes. As a hint, at low temperatures we obtained improvements in the final composition of important compounds, such as higher glycerol contents and a lower ethanol yield, as well as the better fermentation performance of some yeast combinations, especially those of the *S. cerevisiae* with cryotolerant SNC species.

## Author contributions

JA, EB, and AQ conceived and designed the experiments. JA and ML performed the experiments. JA, EB, and AQ analyzed the data and wrote the paper.

## Funding

JA was supported by a FPI grant from the Ministerio de Economía y Competitividad (ref. BES-2013-066434). This work was supported by grants AGL2012-39937-C02-01 and AGL2015-67504-C3-1-R from the Spanish Government and FEDER to AQ, AGL2012-39937-C02-02 and AGL2015-67504-C3-3-R from the Spanish Government and FEDER to EB, and PROMETEO (project PROMETEOII/2014/042) from Generalitat Valenciana to AQ. We acknowledge support of the publication fee by the CSIC Open Access Publication Support Initiative through its Unit of Information Resources for Research (URICI).

### Conflict of interest statement

The authors declare that the research was conducted in the absence of any commercial or financial relationships that could be construed as a potential conflict of interest.
