# Supplementary material for: Effect of Temperature on the Prevalence of Saccharomyces Non cerevisiae Species against a S. cerevisiae Wine Strain in Wine Fermentation: Competition, Physiological Fitness, and Influence in Final Wine Composition
Source: Front Microbiol. 2017 Feb 7;8:150. doi: 10.3389/fmicb.2017.00150 (PMC5293751; doi:10.3389/fmicb.2017.00150)
Supplement: Supplementary file 1 [file Table1.docx]

Effect of Temperature on the Prevalence of *Saccharomyces* Non *cerevisiae* Species against a *S. cerevisiae* Wine Strain in Wine Fermentation: Competition, Physiological Fitness and Influence in Final Wine Composition

Javier Alonso-del-Real^1^, María Lairón-Peris^1,2^, Eladio Barrio^1,2^, Amparo Querol^1*^

^1^Food Biotechnology Department, Systems Biology in Yeast of Biotechnological Interest, Instituto de Agroquímica y Tecnología de los Alimentos, IATA-CSIC, Valencia, Spain

^2^Departament de Genètica, Universitat de València, València, Spain

***Correspondence:** Corresponding Author: jararias04@gmail.com

## Supplementary Table 1: List of primers used for qPCR experiments according to their target strains. The field *Group* indicates which primers have been used together in our qPCR reactions due to lack of cross amplification between them.

| **Primer** | **Target strains** | **Group** | **Sequence (5' → 3')** |
| --- | --- | --- | --- |
| Sc-F | *S. cerevisiae* T73 | A | CGATTTCGATGCTACTCACG |
| Sc-R | *S. cerevisiae* T73 | A | TTATCGCCTGATGGACTGTC |
| Sk-F | *S. kudriavzevii* CR85 | A/B | AATTTAGGTGCCACCCACG |
| Sk-R | *S. kudriavzevii* CR85 | A/B | TTATCTGCCGGTGAACTCAC |
| Su-F | *S. uvarum* BMV58/CECT12600 | A | ACAACCGTATAGTGGCAGG |
| Su-R | *S. uvarum* BMV58/CECT12600 | A | AATCTTCTCACAACGGTGGC |
| Sc-F2 | *S. cerevisiae* T73 | B | GATTTCGATGCTACTCACGAG |
| Sc-R2 | *S. cerevisiae* T73/YPS128 | B | TTACTATTATCGCCTGATGGAC |
| ScYPS128-F | *S. cerevisiae* YPS128 | B | CTGATTTCGATGCTACTCACG |
| Se-F | *S. eubayanus* NPCC1292 | B | AGACCGGCTGATCTACTGG |
| Se-R | *S. eubayanus* NPCC1292 | B | GACGCTACTTTGATGTCATCC |
| Sp-F | *S. paradoxus* 54 | B | GAAGACGACGGAATCATCAC |
| Sp-R | *S. paradoxus* 54 | B | TCACCAGTCAGAATTGCAGG |
